# The stepped wedge cluster randomised trial: an opportunity to increase the quality of evaluations of service delivery and public policy interventions

**DOI:** 10.1186/1745-6215-16-S2-P4

**Published:** 2015-11-16

**Authors:** Monica Taljaard, Richard Lilford, Alan Girling, Karla Hemming

**Affiliations:** 1 University of Ottawa, Ottawa, Canada; 2 University of Warwick, Coventry, UK; 3 University of Birmingham, Birmingham, UK

## Background

The stepped wedge cluster randomised trial (SW-CRT) is a novel research study design that is increasingly being used in the evaluation of service delivery type interventions. The design involves random and sequential crossover of clusters from control to intervention, until all clusters are exposed.

## Aims

We illustrate the use of the design by giving case examples, summarise the results of an update of a methodological systematic review of the quality of reporting and provide recommendations for reporting and analysis.

## Methods

A methodological systematic review of published SW-CRTs. Assessment was guided by recent developments in statistical design and analysis of these studies.

## Results

The use of the SW-CRT is rapidly increasing and that areas of use are diverse. We illustrate how the design is being used to evaluate the effectiveness of a complex intervention, being rolled-out across 90 UK hospitals, to reduce mortality in patients undergoing emergency laparotomy.

Quality of reporting is found to be low. In a SW-CRT more clusters are exposed to the intervention towards the end of the study than in its early stages. A result which *prima facia* might look to be suggestive of an effect of the intervention may therefore transpire to be the result of a positive underlying temporal trend.

A large number of studies do not report how they allowed for temporal trends in the design or analysis.

## Conclusions

The SW-CRT is a pragmatic study design which can reconcile the need for robust evaluations with political or logistical constraints. Quality and reporting is generally low and so consensus guidelines on reporting and analysis are urgently needed.

